# Evaluating the mental health awareness of stakeholders in cycling teams: Results from a cross-sectional survey

**DOI:** 10.1192/j.eurpsy.2025.1881

**Published:** 2025-08-26

**Authors:** A. Smith, J. Grana, J. Colangelo, A. Buadze, M. Liebrenz

**Affiliations:** 1 Department of Forensic Psychiatry, University of Bern, Bern; 2 Psychiatric University Hospital, University of Zurich, Zurich, Switzerland

## Abstract

**Introduction:**

Sports psychiatry is a developing subdiscipline, which emphasises the need for adequate treatment and prevention schemes to uphold the mental wellbeing of athletes. Previous studies indicate that elite-level male cyclists face distinctive socioenvironmental risk factors, including external pressures from teams, particularly in relation to weight management concerns. However, there has been little attention to the mental health support available within teams and the awareness of relevant stakeholders to psychiatric issues.

**Objectives:**

This study sought to gain perspectives on the level of mental health awareness from stakeholders in elite-level cycling teams (i.e., sporting directors, coaches, and medical staff ).

**Methods:**

An anonymous online survey has been compiled containing quantitative and qualitative questions for sporting directors, coaches, or medical staff about their own mental health awareness and literacy. This was distributed to cycling teams and through a national-level federation. The survey link will be available online from the beginning of October 2024 to the end of February 2025. An ethical application was made to the Ethics Committee in the Canton of Bern, who determined that the research fell outside the scope of the Swiss Human Research Act and therefore did not require formal approval.

**Results:**

Preliminary results will be ready in March 2025 in time for the poster display at the European Congress of Psychiatry in April 2025. The findings will provide insights into mental health awareness amongst team stakeholders in men’s elite-level cycling. The quantitative data will be studied with descriptive statistics and the qualitative results will be evaluated using thematic content analysis to identify key themes.

**Conclusions:**

Based on prior literature, our hypotheses are that there is limited mental health awareness about the mental health of elite-level riders amongst cycling team stakeholders and scarce knowledge about how to manage these issues should they arise. These findings would underline a need for more attention to this topic within the sport, potentially necessitating the involvement of national federations and regulators.

**Disclosure of Interest:**

A. Smith: None Declared, J. Grana: None Declared, J. Colangelo: None Declared, A. Buadze: None Declared, M. Liebrenz Consultant of: In cooperation with Swiss Cycling, Michael Liebrenz provides mental health support to elite-level riders involved with the Swiss national team.

